# *Cuniculiplasmataceae*, their ecogenomic and metabolic patterns, and interactions with ‘ARMAN’

**DOI:** 10.1007/s00792-018-1071-2

**Published:** 2018-11-29

**Authors:** Olga V. Golyshina, Rafael Bargiela, Peter N. Golyshin

**Affiliations:** School of Natural Sciences, Bangor University, Deiniol Road, Bangor, LL57 2UW UK

**Keywords:** Acidophilic archaea, *Thermoplasmatales*, *Cuniculiplasmataceae*, *Cuniculiplasma*, *Ca*. Micrarchaeota, ARMAN

## Abstract

Recently, the order *Thermoplasmatales* was expanded through the cultivation and description of species *Cuniculiplasma divulgatum* and corresponding family *Cuniculiplasmataceae*. Initially isolated from acidic streamers, signatures of these archaea were ubiquitously found in various low-pH settings. Eight genomes with various levels of completeness are currently available, all of which exhibit very high sequence identities and genomic conservation. Co-existence of *Cuniculiplasmataceae* with archaeal Richmond Mine acidophilic nanoorganisms (‘ARMAN’)-related archaea representing an intriguing group within the “microbial dark matter” suggests their common fundamental environmental strategy and metabolic networking. The specific case of “*Candidatus* Mancarchaeum acidiphilum” Mia14 phylogenetically affiliated with “*Ca*. Micrarchaeota” from the superphylum “*Ca*. Diapherotrites” along with the presence of other representatives of ‘DPANN’ with significantly reduced genomes points at a high probability of close interactions between the latter and various *Thermoplasmatales* abundant in situ. This review critically assesses our knowledge on specific functional role and potential of the members of *Cuniculiplasmataceae* abundant in acidophilic microbiomes through the analysis of distribution, physiological and genomic patterns, and their interactions with ‘ARMAN’-related archaea.

**Electronic supplementary material:**

The online version of this article (10.1007/s00792-018-1071-2) contains supplementary material, which is available to authorized users.

## Introduction

The history of the order *Thermoplasmatales* had started in 1970 with the discovery of *Thermoplasma acidophilum* when *Mycoplasma*-like cell wall-deficient organism from coal refuse pile was isolated and described (Darland et al. 1970). Noteworthy, in hindsight, this organism appeared to be the first isolated heterotrophic acidophilic archaeon; however, its affiliation with the ‘Third Domain of Life” was recognised much later. Heterotrophic archaea of the order *Thermoplasmatales* are broadly distributed geographically and play an important role in carbon cycling in acidic mine drainage (AMD)-impacted and other low-pH natural environments (Golyshina 2011; Justice et al. 2012). Nevertheless, up to now, the iron oxidation ability was confirmed only for *Ferroplasmaceae* members (Golyshina et al. 2000; Golyshina 2014). Another feature, characteristic of all the studied *Thermoplasmatales,* with an exception for organisms of the genus *Picrophilus,* is the lack of cell walls. Currently, the order *Thermoplasmatales* embraces the most acidophilic organisms described so far and includes six genera with the genus *Cuniculiplasma* being the most recent member to join the club (Golyshina et al. 2016a). Culture-independent metagenomic studies suggested a significant proportion of archaeal inhabitants of low-pH emplacements affiliated with archaea referred to as “Alphabet plasmas” (Baker and Banfield 2003; Kato et al. 2011; Méndez-García et al. 2014, 2015), whose metabolism and morphology remain unclear and could only be suggested by bioinformatic predictions from metagenomic data. One of these “Alphabet plasmas”, designated as “G-plasma”, was detected in relatively higher quantities in microbial communities and its genome was almost fully assembled (Tyson et al. 2004; Yelton et al. 2013). From the study of isolates of *Cuniculiplasma* spp. it became apparent that 16S rRNA and genome sequences of both strains of *Cuniculiplasma divulgatum* isolated from sites located > 1000 km apart exhibited 100% of 16S rRNA gene sequence identity and > 98% average nucleotide identity (ANI) of genomes with the “G-plasma” metagenomic assembly from Iron Mountain (California, USA) (Tyson et al. 2004; Golyshina et al. 2016a, b), suggesting their affiliation with the same species. Other acidophilic community members detected in a lower abundance in acidic environments represent the so-called ‘ARMAN’ group (Baker et al. 2006). One of the representatives of this group, namely “*Candidatus* Mancarchaeum acidiphilum”, related to the members of ‘ARMAN-2’ cluster was shown recently to form an intimate interaction with *Cuniculiplasma divulgatum* strain PM4 (Golyshina et al. 2017b). The ecophysiological patterns of *Cuniculiplasmataceae,* geographic distribution, their genomic signatures and their interconnections with ‘ARMAN’-like community members are reviewed in this paper.

## Biogeography

Two strains of newly proposed *Cuniculiplasma divulgatum* were isolated from mine-impacted environments in Europe, namely Cantareras, Spain and Parys Mountain/Mynydd Parys, Wales, UK (Golyshina et al. 2016a).

These organisms were first detected in metagenomic data in microbial biofilms from Richmond Mine AMD system, with pH about 1 and temperature about 38–42 °C (Iron Mountain, California, USA) and were dubbed as “G-plasma”; later, the genomic signatures of similar organisms were identified in other acidic sites worldwide (see Tyson et al. 2004 and many others). “G-plasma” (*Cuniculiplasmataceae*)-related reads were shown to account for up to 22% of total community proteome in this environment (Richmond Mine, California, USA).

**Fig. 1:**
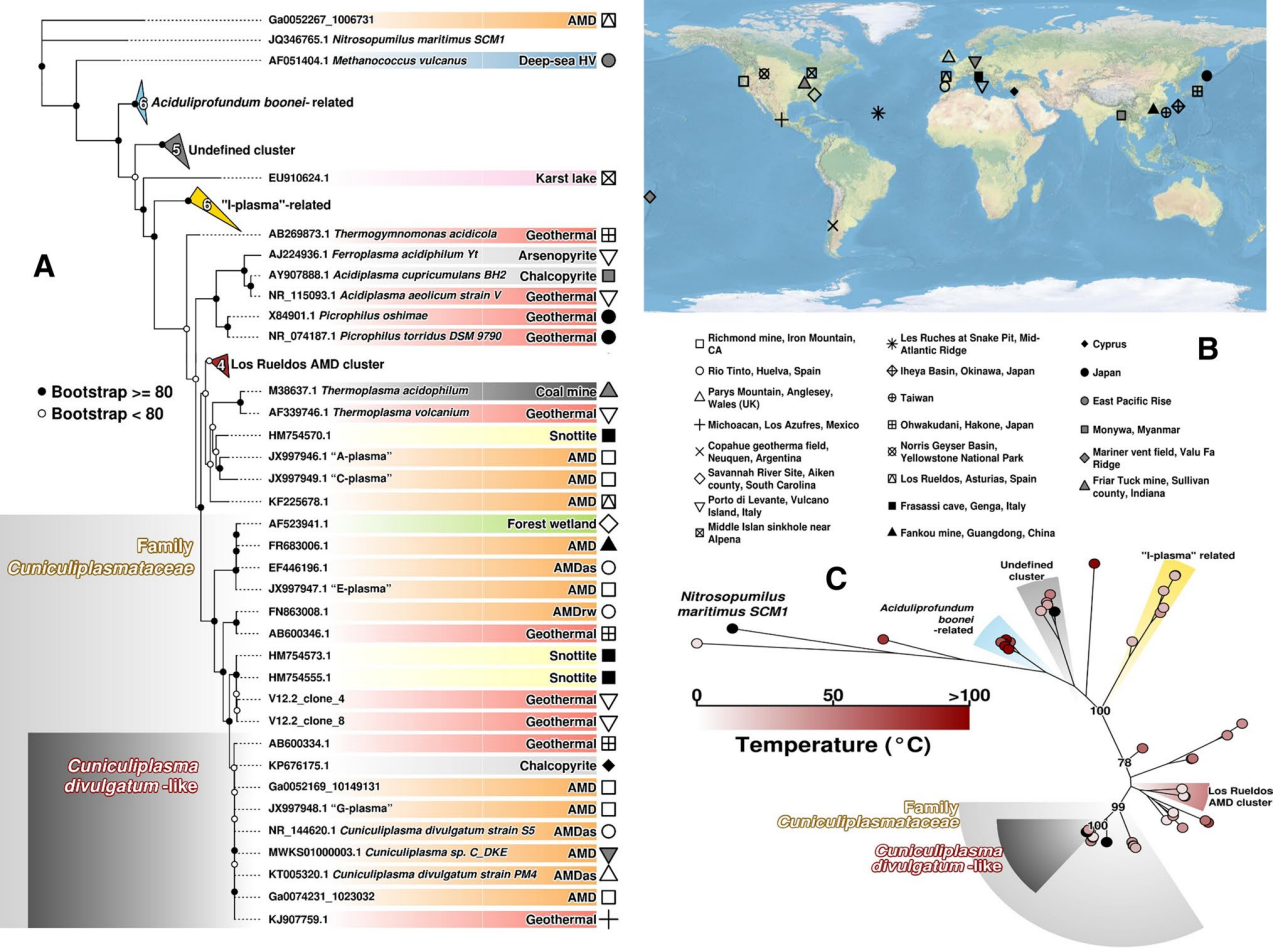
Phylogenetic and geographical distribution of *Thermoplasmatales*. 56 rDNA sequences have been used to develop a maximum likelihood phylogenetic tree based on GTR + I + G model, represented in **a** and **c**. **a** The tree showing accession numbers, an affiliation (where it is possible) and environment type (in different colour background). **b** The geographical location of the isolation sites for the 56 rDNA sequences. Symbols representing each location are also shown in **a**, on the right margin. Temperature range in the moment of isolation is represented in **c** for each sequence of the tree. All figures have been developed under R programing environment using the basic R and the packages *OpenStreetMaps*, *ape* and *phytools*. *Deep-sea HV* deep-sea hydrothermal vent, *AMD* acid mine drainage, *AMDas* AMD acidic stream, *AMDrw* AMD river water

In Frasassi Cave system (Italy) characterised by constant temperatures and pH (13 °C and pH 0–1), “G-plasma”-related archaea contributed approximately 15% of total metagenomic reads (Jones et al. 2012). The study recovered that this particular single phylotype constituted 26 of 28 clonal sequences (Jones et al. 2012). Jones et al. (2012) noticed that 1392r, 1492r and A21f primers showed some mismatches with sequences of this group of archaea; however, it contradicts with our results on successfully amplified 16S rRNA sequences of *Cuniculiplasmataceae* with A21f/1492r primers from environmental samples (Golyshina et al. 2016a, b). Moreover, our census of sequencing data emphasised the presence of these family members in further habitats, such as geothermal areas, cave systems and sulphide ore deposits across continents (Fig. 1, Table S1). For instance, *Cuniculiplasma*-related organisms inhabited a mat sampled from acidic (28 °C and pH 2.5) spring field in Owakudani, Hakone, Japan (Kato et al. 2011). Another example is represented by chalcedonic sinters sampled from Yellowstone’s Norris Geyser Basin heated by geothermal activity to 35 °C with pH 1 (Walker et al. 2005). The two latter examples of geothermal habitats suggest that *Cuniculiplasma*-related organisms do rather favour moderate-temperature niches. On the other hand, *Cuniculiplasma*-related signatures were also discovered in metagenomes from hot spring mat sampled in Los Azufres National Park of Mexico, with measured temperature of 73.4 °C and pH of 3.8 (Chen et al. 2018).

A closer look at the scattering patterns of *Cuniculiplasma* spp. in AMD systems revealed their preferences for biofilms and, to a lesser extent, for sediments and surface water samples (Fig. 1).

Both *Cuniculiplasma* strains were isolated from streamers (green-coloured filaments) formed by microalgae associated with AMD systems from Cantareras, Iberian Pyrite Belt, Spain, and Parys Mountain, UK, which run through copper sulphidic deposits (Golyshina et al. 2016a). During warmer seasons, water surfaces and acid streamers’ microniches may warm up to higher temperatures, compared to sediments. The overall temperature regime in such environments is suitable for mesophilic, potentially thermotolerant microorganisms; however, some appearance of thermophilic counterparts cannot be excluded, either. On the other hand, seasonal low temperatures in both AMD environments point at some degree of psychrotolerance, which was, in fact, confirmed by the temperature growth profiles established for both *Cuniculiplasma divulgatum* strains (Golyshina et al. 2016a). Furthermore, *C. divulgatum*-related signatures were detected by Ziegler et al. (2013) in samples of snottite biofilms collected from the abandoned pyrite mine in the Harz Mountains in Germany. Localisation of archaea in this ecological niche was perceived to be connected exclusively to anoxic areas, and a common fundamental strategy was suggested for those organisms to rely on metabolic intermediates and end products leaked by other organisms inhabiting the same environment (Ziegler et al. 2013). These points are definitely valid for *C. divulgatum,* which is as experimentally revealed, facultatively anaerobic and heterotrophic organism relying on an external input of organic compounds that it is unable to synthesise (Golyshina et al. 2016a). Furthermore, Krause et al. (2017) produced a stable enrichment culture after 2.5 years of cultivation from that particular sample consisting of four counterparts, including *C. divulgatum*-related organism, as revealed by metagenomic, metatranscriptomic analysis and catalysed reporter deposition fluorescence in situ hybridization (CARD-FISH) visualisation.

*Cuniculiplasmataceae* were furthermore detected in sampling spots with lowest dissolved oxygen concentrations of Los Rueldos AMD system described as a stagnant streamer-like microbial mat characterised by pH about 2 and temperatures ranging between 10 and 17 °C. Redox potential and conductivities within this system were determined as ~ 256 mV and 5.14–6.72 mS cm^−1^, respectively, found to be amongst the lowest values for AMD (Méndez-García et al. 2014). In addition, *Cuniculiplasma* spp. along with *Ferroplasma* spp. were found in 16S rRNA PCR amplicon reads from enrichment cultures established with Los Rueldos acid streamer samples (Golyshina et al., unpublished).

Short fragments of *Cuniculiplasma*-related sequences in V3–V4 PCR amplicons were recovered from Copper Cliff AMD pond (SRS404029, Ontario, Canada) and from Southwest China, Guiyang, in sites affected by AMD: soil (SRX1438201) and watershed (SRX655594). Furthermore, in metagenomes from three mining sites in Guangdong province (China) characterised by pH 0.83–2.74, *Cuniculiplasma*-related archaea (SRX079112, SRX079111 and SRX079110) were found (Chen et al. 2018).

In summary, the archaea of the family *Cuniculiplasmataceae* are ubiquitously present in natural or man-made ecosystems with low and extremely low pH and are previously neglected acidophilic cosmopolites (Golyshina et al. 2016b).

The analysis of *Cuniculiplasmataceae*-derived sequencing data identified their rather wide distribution in relation with habitat temperatures, with a preference to mesophilic growth conditions (Fig. 1). The origins of the data are mostly limited to the northern hemisphere, and it seems feasible that the further species and genera do exist within this family. Thus, based on the phylogeny of 16S rRNA gene sequences we suggest the existence of another, in addition to *C. divulgatum,* species within the genus *Cuniculiplasma* and probably another genus within the family *Cuniculiplasmataceae* (Fig. 1). Since the 16S rRNA gene sequence identity level above 90% cut-off suggests organisms belong to the same family, we consider the numerous uncultured organisms clustering together with *C. divulgatum* strains above that threshold form the family *Cuniculiplasmataceae*. “G-plasma” cluster is, therefore, equivalent to a validly published taxon, the family *Cuniculiplasmataceae,* and the original ‘G-plasma’ metagenomic assembly (Tyson et al., 2004) was derived from the organism of the species *C. divulgatum,* sharing > 97% of average nucleotide identity (ANI) with the type strain *C. divulgatum* S5.

## Genomic diversity and conservation

Currently, altogether eight genomes of *Cuniciliplasma*-related organisms from Eurasia, South and North America are available. The first metagenomic assembly of ‘G-plasma’ was done by Tyson et al. (2004) and furthermore analysed by Yelton et al. (2013). The only complete (ungapped) genomes from this group of organisms were produced for strains S5 and PM4 of *C. divulgatum* by Golyshina et al. (2016b). The genome of C_DKE variant (100% identity with 16S rRNA genes with both *C. divulgatum* strains) was recovered from the enrichment culture (Krause et al. 2017). All the above genomes exhibited extremely high similarities despite various degrees of their completeness (Chen et al. 2018). Additionally, genomes of *Cuniculiplasma*-related species were retrieved from metagenomes from AMD (Fankou, China), from two geothermal areas located in Tengchong, China, and in Los Azufres National Park (Mexico) and from an enrichment culture established with samples from Yunfu Pyrite Mine (China) (Chen et al. 2018). Comparison of genomes of *C. divulgatum* strains S5 and PM4 and of ‘G-plasma’ showed average nucleotide identity (ANI) between both isolates S5 and PM4 at 98.8%. ‘G-plasma’ genome showed ANI values of 98.7% with S5 and 98.4% with PM4 strains suggesting affiliation of all organisms to the same species, considering the generally accepted ANI threshold for same species being > 95%. High levels of average amino acid identity (AAI) were identified as well, 98.84% between both strains and 98.37% and 98.4% between “G-plasma” variant and strains S5 and PM4, respectively. Moreover, 1174 protein groups comprised the core in silico proteome of *C. divulgatum* strains and ‘G-plasma’ (Golyshina et al. 2016a, b).

Comparison of *C. divulgatum* genome with C_DKE variant from enrichment culture, established from a snottite sample from Harz Mountains, Germany, has been reported by Krause et al. (2017). Altogether, 1426 gene families were found to be present in both the indicated genomes; however, 80 and 445 families were revealed to be unique for *C. divulgatum* S5 and to C_DKE metagenomic assembly (the completeness of the latter estimated as 86%), correspondingly. In this work, the enrichment culture from Harz Mountains sample was grown under anaerobic conditions at lower temperature (22 °C), which probably explains its slower growth in laboratory (Krause et al. 2017), as compared with the strains S5 and PM4, whose mesophilic and facultatively anaerobic and heterotrophic lifestyles have been shown through cultivation experiments (Golyshina et al. 2016a). *Cuniculiplasmataceae* archaea, like all extreme acidophiles, flourish in ecosystems characterised by acidic pH, high concentrations of metals or metalloids. As heterotrophs, *Cuniculiplasmataceae* rely on organic compounds as carbon and energy sources. The origin of organic carbon in their natural habitats could be from dead cells, a from exudates from metabolising cells, or from primary producers, such as acidophilic algae; indeed, both *Cuniculiplasma* strains were isolated from environments containing high proportion of algae, whose metabolites could be substantial to support the life of these archaea.

Up to now, *Cuniculiplasma divulgatum,* together with archaea of the family *Ferroplasmaceae,* are the only cultivated archaeal representatives from moderate- and low-temperature acidic sites. Iron oxidation, which is considered as a highly possible metabolic feature for inhabitants of ferrous and ferric-rich environments, was not confirmed for *Cuniculiplasma divulgatum* strains; this trait is still exclusively found in archaea of the family *Ferroplasmaceae* (Golyshina 2014; Golyshina et al. 2017a). Regarding the iron oxidation, some points need to be emphasised here. Genes encoding homologs of rusticyanin or sulfocyanin are often associated with GI (Genomic Islands) in *Thermoplasmatales*, as shown for *Picrophilus torridus, Ferroplasma acidiphilum* and *Cuniculiplasma divulgatum* and could be transmitted via the lateral gene transfer (Fütterer et al. 2004; Golyshina et al. 2017a, and also please see below). More importantly, the presence of these genes in genomes is not necessarily an indication of iron oxidation capacity, as show the examples of some *Sulfolobus* spp., *Picrophilus torridus* or *Cuniculiplasma divulgatum* (Fütterer et al. 2004; Golyshina et al. 2016a, b).

The genomic analysis revealed particular compelling genomic features found for these organisms (Golyshina et al. 2016b). *Cuniculiplasma* species were considered to possess an ancestral form of A-type terminal oxygen reductase/A1-type heme–copper oxidases forming a distinct clade, located near the B-type oxygen reductases and the root of all the other A-type reductases (Golyshina et al. 2016b). The analysis of central metabolic processes of *Cuniculiplasma* spp. delineated key mechanisms and fundamental principles for archaea of the order *Thermoplasmatales* (Golyshina et al. 2016b; Krause et al. 2017). There was an absence in both genomes of entire pathways for biosynthesis of several amino acids and the presence of peptidases, peptide/amino acids transporters (Golyshina et al. 2016b). This modulates and controls the important mode of action and impact on the microbiota influenced by dependence on external sources of these biomolecules and, therefore, determines the specific role of these archaea as scavengers of complex organic compounds provided by their microbial neighbourhood. Interesting is also the remarkable conservation within GI for these three genomes derived likely from other euryarchaea populating the very same environments. This persistent flow of particular genetic pool’s counterparts appears as an important part of adaptive strategy of these organisms. The GIs bear genes for toxin–antitoxin, restriction–modification systems and metal-, efflux-, transport-, and oxidative stress response-related proteins (Golyshina et al. 2016b). These ‘defence’ islands facilitate a prompt resilience and adaptation of these archaea to the potential viral attack and high concentration of metals. Furthermore, *Cuniculiplasma* genomes encode the clustered regularly interspaced short palindromic repeats (CRISPR)-Cas defence systems. About 9% of all palindromic repeats in the genome *C. divulgatum* PM4 and 7% of those in strain S5 showed 90–100% sequence identity with microbial and viral sequences in Richmond Mine, pointing at the existence of common viruses in all three acidic ecosystems (Golyshina et al. 2016b). Nevertheless, up to now no archaeal viruses were isolated from mesophilic acidic environments (Krupovic et al. 2018). Two types of viruses of spindle and rod shape associated with some small cells considered to be ‘ARMAN’ related have been recognised by electron microscopy from Iron Mountain AMD site (California, USA), though no information on taxonomic affiliation of those was presented (Comolli et al. 2009). Metagenomic survey of Andersson and Banfield (2008) suggested the presence of proviruses in the virosphere in the above site. For some viral types, connectivity was predicted for rather immense host range among *Thermoplasmatales*-related archaea represented by metagenomic variants, and for some viruses to be linked to singular community members only (Andersson and Banfield 2008). A further research into the viruses specific to the mesophilic acidophilic archaea requires more attention to shed some light on these, currently neglected, drivers shaping the composition of acidophilic microbial communities.

## Interconnection of Cuniculiplasmas with ‘ARMAN’ archaea

In the process of isolation of *Cuniculiplasma divulgatum* strain PM4, its co-culturing ‘ARMAN’-2-like organism, “*Candidatus* Mancarchaeum acidiphilum” Mia14 was reported (Golyshina et al. 2017b). “*Ca.* Mancarchaeum acidiphilum” belongs to ‘ARMAN’ cluster. The presence of these deeply branching lineages in acidic samples from Iron Mountain (CA, USA) together with numerous uncultured *Thermoplasmatales* archaea was discovered by shotgun sequencing (Baker et al. 2006, 2010). Represented in minor quantities in environmental settings, these filterable archaea belong to the “microbial dark matter” and lack functional characterisation. “*Candidate* Micrarchaeota” and “*Ca.* Parvarchaeota” reflecting ‘ARMAN’ diversity were proposed and both candidate phyla were later included into the ‘DPANN’ ‘superphylum’ (Rinke et al. 2013). However, it remained unclear which archaea of the order *Thermoplasmatales* could serve as hosts for ‘ARMAN’ and what is the level of interdependency between these organisms.

The analysis of the co-culture of *C. divulgatum*, strain PM4 with “*Ca*. Mancarchaeum acidiphilum” (ca. 10% genomic reads or 20% or the total population) revealed that the ungapped genome of Mia14 (the only completed genome of ‘ARMAN’-related organism up to now) lacked main metabolic pathways, pointing at reliance on host metabolites. TCA cycle, glycolysis, and other central pathways were either absent or incomplete in the genomic blueprint of Mia14 (Golyshina et al. 2017b). Furthermore, many metabolic precursors (amino acids, cofactors, nucleotides and others) were not found to be encoded by the genome. A puzzling issue is related to the ability of independent respiration in Mia14; while all genes coding for cytochrome *bd* quinol oxidase were identified, its genome is completely devoid in any genetic loci for the synthesis of isoprenoid quinones, the only electron donors for this enzyme complex. In this regard, the utilisation by Mia14 of *Cuniculiplasma* membrane quinones originated from living or dead cells has been proposed as the only feasible explanation (Golyshina et al. 2017b). The reduced genome size (0.95 Mbp) together with limited metabolic capability considered its “ectoparasitic” lifestyle and dependence on *C. divulgatum* PM4. Derived from the genome analysis, cultivation settings and fluorescence microscopy data, the conclusion was made that the external supplement of proteinaceous substrates and amino acids may also be essential for the growth of Mia14. CARD-FISH data suggested that cells of Mia14 were of a greater sizes than generally predicted for ‘ARMAN’, significantly exceeding the diameter of membrane filter pores (0.22 μm), the same observation was done early for other organisms of the order *Thermoplasmatales* (Golyshina et al. 2017b). Comparative genomic analysis of *Cuniculiplasma* isolates PM4 and S5 and shotgun sequence data of samples from Parys Mountain indicated significant genes’ flow in situ between Mia14 and its putative host. Among organisms of ‘DPANN’ ‘superphylum’, Mia14 experienced the biggest gene loss while evolving from its closest ancestor.

It is worth noting the presence in the genome of Mia14 of two recognisable type IV pili systems and a number of genes encoding glycoprotein (laminin-like), linked to the formation of extracellular matrix, important in cell-to-cell interactions. Some of these laminin-coding genes are located in a close proximity to type IV pili genes and within a GI. Altogether, the genome analysis and laboratory experiments suggested *Cuniculiplasma* spp. as an essential host to support the lifestyle of Mia14 archaeon, whereby diverse systems, such as membrane channels, surface proteins and pili may facilitate the exchange of macromolecules and their precursors, and DNA transfer between these two organisms (Golyshina et al. 2017b).

In a similar independent study, the enrichment culture established with snottite sample from Harz Mountains was shown to harbour four organisms, including one related to ‘ARMAN’-1. Besides this organism, the consortium consisted of two *Thermoplasmatales* (one of which was a B_DKE related to *Thermogymnomonas acidicola* and *Cuniculiplasma divulgatum*, with the SSU rRNA gene sequence identity of 91.6%, and 91.7%, correspondingly, and another, C_DKE, presented in minor quantities only, with 100% SSU rRNA gene sequence identity to *C. divulgatum*) and a fungus. CARD-FISH data suggested associations of ‘ARMAN’ cells with both *Thermoplasmatales* (Krause et al. 2017).

Chen et al. (2018) discussed the metabolic potential of ‘ARMAN’ archaea, their biogeographical patterns and evolutionary history. Authors suggested ‘ARMAN’ have some deficiencies in biosynthetic pathways for amino acids and nucleotides, similar evolutionary patterns of energy-generating metabolic pathways, and potential dependency of *Thermoplasmatales* in situ. At the same time, the study suggested very versatile catabolic abilities in ‘ARMAN’ towards various organic substrates, which are more typical for free-living organisms and somewhat contradict the hypothesis of ectoparasitism and strict dependency on the host. The wide distribution of ‘ARMAN’-related signatures in diverse environments advocates that these archaea might live in close proximity to numerous distinct *Thermoplasmatales* organisms (Chen et al. 2018).

## Conclusion

The high relative abundance of *Cuniculiplasmataceae* in hyperacidic environments and their global ubiquity point at their functional importance and substantial contribution to the processes of element cycling in these habitats, though cultured isolates are still merely represented in the family. The first insights into their metabolism and lifestyles were gained thanks to the successful isolation of the two strains and their consequent experimental physiological studies supported by the analyses of the first two high-quality, ungapped genomes representing this family. A high level of conservation of genomes in geographically distant habitats, similar adaptive strategies to cope with environmental conditions, acquisition of ‘defence’ genomic islands for antiviral or to high metal concentrations resistance contribute to their success in colonisation of a variety of acidic environments. Their co-existence with ‘ARMAN’-related organisms shown in the laboratory culture and in natural habitat has unambiguously established the full dependency of the latter ‘DPANN’ representatives on particular *Cuniculiplasmataceae* in acidophilic microbiomes. Serving as a host, *Cuniculiplasmataceae* provide key metabolites for growth and energy generation to “*Ca*. Micrarchaeota” and promote environmental survival of these elusive organisms. The success in cultivation of pure and binary cultures will further advance our understanding of interactions and specific roles of acidophilic archaea in extremely low pH environments.

## Electronic supplementary material

Below is the link to the electronic supplementary material.

Supplementary material 1 (XLSX 14 kb)
